# American Medical College Association

**Published:** 1879-05

**Authors:** 


### AMERICAN MEDICAL COLLEGE ASSOCIATION.

Atlanta, May 3, 1879.

The meeting was called to order, at 10 o'clock, by Vice President N. S. Davis, of Chicago, who made some appropriate remarks relative to the life and death of the former president, Professor Biddle of Philadelphia.

The first business in order being the reception of credentials, the Secretary, Professor Connor, made out a list of the following accredited members:

Jefferson Medical College, Philadelphia—Professor S. D. Gross.

Medical Department University of Louisville, Kentucky—Professor Jno. E. Crowe.

Hospital College of Medicine, Louisville—Dr. Robt. L. Breck and Professor D. S. Reynolds.

Medical Department Iowa State University, Iowa City—Professor W. F. Peck.

Chicago Medical College—Professor N. S. Davis.

Detroit Medical College—Professor Laertus Connor.

Starling Medical College, Starling, Ohio—Professor S. Loving.

Medical Department of University of Nashville and Vanderbilt—Professor W. T. Briggs and Thos. Menees.

Atlanta Medical College—Professors J. G. Westmoreland, Taliaferro, et. al.

Kansas City College of Physicians and Surgeons—Professor T. B. Lester.

Miami Medical College, Cincinnatti, Ohio—Professor Jno. A. Murphy.

Louisville Medical College—Professor C. W. Kelly.

Medical Department University Michigan—Professor E. S. Dunster.

Rush Medical College, Illinois—Professor Moses Gunn.

Texas Medical College and Hospital—Professor Greensville Dowell.

Ohio Medical College—Professor W. W. Dawson.

Medical College of South Carolina—Professor J. F. Prioleau and J. P. Cazal.

Medical Department University of Louisiana—Professor S. E. Chaille and E. S. Lewis.

Professor Bodine raised the point as to who should be delegates to the Association; whether delegates chosen from the Boards of Trustees, or not.

This question was dicussed by Professors Gunn, Dunster, Murphy and others; when the President decided that the constitution required that the Board of Trustees should be represented by its executive officer, only. Ruled in this manner from the reason that if the Faculty alone was meant to be represented, the constitutional ambiguity would not have existed.

Upon continuance of the debate, the chair ruled that further discussion was out of order.

At this stage, certain delegates who represented Boards of Trustees, having been declared ineligible, were by motion, allowed a seat in the meeting during its session.

Reading the minutes being next in order, it was dispensed with on motion.

The next order of business being the consideration of the amendment to the By-Laws and Constitution offered by Bellevue Hospital Medical College, and endorsed by College of Physicians and Surgeons, New York, at last year's meeting.

Dr. Dunster moved to take them from the table and put them on their final passage.

Dr. Reynolds offered an amendment to the effect that it should be made five per cent. of graduates, instead of ten, which amendment was lost.

The amendment to Section 5, Article V, of the Articles of confederation of the American Medical College Association: Instead of "five per cent. of the number of matriculates," read "ten per cent. of the number of graduates," being the original question, was put and lost by a vote of ayes ten, nays six, the necessary two-thirds not being given in favor.

Dr. Briggs moved that as the application of the Atlanta Medical College had been presented, but too late by a few days only, to allow its delegates a seat in the meeting, that their credentials be accepted and they be allowed membership. Motion carried.

The proposed amendment to the Constitution for an additional section to Article VII, was agreed to by a vote of ayes thirteen to nayes one.

The amendment proposed by Dr. Menees at the last meeting of the Association, was agreed to by ayes eleven nays two.

Next in order, was the consideration of the charges and specifications made at the last meeting of the Association, against the Louisville Medical College and the Kentucky School of Medicine.

The Chair explained that those charges could not now be entertained, because first, they were made by a committee and not by a member, as required by the constitution; and, second, the accusers, the Bellevue Medical College, were absent from the meeting.

Dr. Dunster thought that it was but justice that some action should be taken, as the accused, if innocent, should be exonerated, and if guilty, condemned.

The Chair ruled, that any motion to consider the charges, was out of order.

Next in order, was the consideration of the report of the special committee, appointed last year, on the registration of Colleges, composed as follows; Drs. A. Flint, Jr., S. D. Gross and N. S. Davis. Upon motion, the report was read, received and its consideration postponed to afternoon session.

Association adjourned till 3 o'clock, P. M.

AFTERNOON SESSION.

Pursuant to adjournment, the President called the meeting to order at 3 o'clock. On motion, the reading of minutes was dispensed with.

Dr. Murphy moved that the report of the special committee on Registration of Colleges be re-committed to the original committee for correction and condensation.

Drs. Dawson, Gross and Dunster discussed the injustice of neglecting the settlement of the charges the report of which makes against certain schools.

Dr. Chaille amended the resolution to re-commit and report again next meeting, by suggesting that the corrected report be presented as soon as practicable for publication.

Dr. Murphy thought that this would place the disposition of the report with the Secretary, as the Association would not have opportunity of considering it until after its official publication.

Dr. Reynolds offered as a substitute, a motion to refer the report to a committee of seven, with instruction to correct certain inaccuracies and report on the following Monday.

Dr. Dawson suggested as an amendment to Dr. R's substitute, that as many of the original committee as were present, be added to the committee of seven. Accepted by Dr. Reynolds.

The amendments and substitutes being rejected, the original resolution was put to vote and carried.

Dr. Murphy moved the appointment by the Chair, of a committee on the registration of Colleges, as suggested in the report of the special committee, whose report has been read and re-committed for correction. Motion being carried, the President appointed Drs. Connor, Dunster and Murphy.

The annual reports of members were read by the Secretary, which showed a membership of twenty-eight Schools, with four applications pending, and one affiliated College.

Dr. Bodine moved that the St. Joseph College be permitted to withdraw its application for membership, pending the vote on which discussion was had as to the expediency of permitting such withdrawal. Finally, the motion being put on its passage, was carried.

Next in order, was permitting Dartmouth College to withdraw from membership. Motion to permit the withdrawal was passed.

Dr. Bodine offered the following resolution:

*Resolved*, That it is unlawful for credit to be granted to any student for two regular courses when the same are taken within less than fifteen months. Passed.

Election of officers being next in order, a motion to postpone the election till next monday, was carried.

Dr. Bodine offered the following reolution:

*Resolved*, That gratuitous instruction of medical students, by any member of this Association, otherwise than provided for in Sections 3, 4 and 5, Article V, of the Articles of Confederation, is unlawful.

Upon discussion of the resolutions, by Drs. Murphy, Kelly of Louisville, Cook, Dunster and others, it was finally adopted.

Dr. Peck moved that a committee of three be appointed by the President, to draught a body of resolutions appropriate to the memory of the lamented Dr. Biddle, former President of this Association. Motion was carried, and Drs. Dawson, Gross and Gunn appointed on the committee.

Dr. Starling Loving, Secretary of the Convention of American Colleges, acting under instructions from that Convention, presented a report of their proceedings. The report was received and made special order of business on Monday.

Dr. Reynolds offered the following resolution:

*Resolved*, That members of this Association, cannot in any way recognize the tickets of any institution which, by reason of insufficient length of lecture course, or insufficient previous period of study, are ineligible to membership in this body. Resolution laid on table till Monday.

Association adjourned to Monday.

---

SECOND DAY'S SESSION—MONDAY, MAY 5TH.

The Association met in the Senate Chamber at 10, A.M., Dr. N. S. Davis presiding.

The minutes of Saturday's proceedings were read, and, with slight corrections, approved.

The next order of business was the election of officers for the ensuing year; but Dr. W. F. Peck asked, as a matter of courtesy and respect, that the resolutions upon the death of the late Dr. Biddle be first read. The resolutions are as follows:

"Since the assembling of this Association death has invaded our circle, and launched his shaft with unerring aim against our presiding officer.

"Professor J. B. Biddle, who presided over the convention which called this Association into existence, and who was twice its President, was widely known as a gentleman of culture and refinement, a teacher who possessed the happy gift of imparting knowledge, a physician of rare ability, and an author whose written wisdom reached and benefitted his professional brethren.

"To us who felt his guiding hand in our deliberations, he was known as an officer of quick perception, clear head, decisive ruling, and winsome urbanity.

"While, then, we are compelled to bow in submission to the stern decree which has removed him from our midst, we cannot refrain from expressing our deep sorrow for our own loss, and tendering to his bereaved family our heartfelt sympathy in this their hour of grief and sorrow.

"Let us honor his memory by emulating his virtues.

W. W. DAWSON,
S. D. GROSS,
MOSES DUNN."

The motion that a copy of these resolutions be spread upon the minutes; that a memorial page be devoted to them in the official report of this Convention, and that a copy be forwarded to the bereaved family, was carried.

The election of officers being next in order, Dr. Samuel D. Gross, of Philadelphia, was nominated for President, and received fifteen votes, a majority of those cast.

Dr. N. S. Davis, of the Chicago Medical College, was nominated for Vice-President, and received fifteen votes, nineteen being the whole number cast. Elected.

Dr. Leartus Conner, of the Detroit Medical College, was elected Secretary and Treasurer, receiving fourteen votes out of seventeen cast.

Next in order was the reading of a communication from the "Convention of Medical Colleges," which held a session in Atlanta, May 2d. At that Convention the questions discussed were:

(1.) Should all Medical Colleges require three regular courses of lectures in three separate years as one of the requirements for conferring the degree of M.D.?

(2.) Should all Medical Colleges require before admitting to matriculation a preliminary examination—such examination embracing at least the elements of the physical sciences in addition to a fair English education?

Since only twenty-three of the fifty-nine Medical Colleges in the United States were represented in that Convention they were powerless to act, and this communication was intended to bring these questions before the present Association where decisive action could be taken upon them. The first of the questions thus referred was disposed of by an amendment to the articles of confederation, proposed by Professor Menees, by which said questions was answered affirmatively. Under the rules of the Association the amendment was tabled till the next session. The second of the referred questions was also laid upon the table for one year.

The following amendment to Article 1 of the Articles of Confederation was then offered by Prof. Bodine: The majority of the members of one faculty shall not constitute the majority of the members of another faculty, unless the sessions of the two schools are held simultaneously.

The amendment was seconded, and under the rules lies over till next year.

Professor Chaille called from the table the following resolution: That it shall be considered derogatory to the dignity and good standing of any medical college represented in this Association to advertise in any other than a strictly medical publication the names of its Professors with their respective Chairs.

This resolution does not apply to the annual circulars and catalogues issued by the colleges, but to advertising non-professional periodicals, newspapers and other like publications in which only a card calling attention to the advantages of the school, length of session, fees, etc., with the names of the executive officer or secretary, should be permitted.

After full discussion by Professors Chaille, Dawson, Murphy, Lankford and others, the resolution was adopted.

The report of the Treasurer, Dr. L. Connor, showing a balance in the treasury of eighty dollars, was then read and approved.

Professor Greensville Dowell offered the following resolution:

That the metric system shall henceforth be used in the minutes of this Association, and in all other papers published under its authority, and that the professors represented in this Association, be requested to teach the metric system in their schools. Laid on the table.

Professor Dunster offered the following amendment to the By-laws: For Section 1, Article V, of the By-Laws, substitute the following:

"Delegates to the meetings of the Association, may be chosen from among the members of the governing boards of a college, or from members of the faculty having a vote upon the graduation of students or from both, but in no case, shall such double representation entitle the college to more than one vote in the Association. Laid over till next year.

On motion of the Secretary, thanks were tendered Professor J. G. Westmoreland and the Georgia State Officials, for providing such comfortable and convenient rooms for the meeting of the Association.

On motion of Dr. Gross thanks were tendered the officers of the Association.

A vote of thanks was tendered to the reporters of the daily papers for their full and accurate reports of the proceedings of the Convention.

It was then moved to adjourn to meet next year in the same place that the American Medical Association meets, and on the Monday preceding the convening of that body.

